# Anomalous kinetic study of atenolol release from ATN@DNA a core-shell like structure

**DOI:** 10.1038/s41598-023-29774-8

**Published:** 2023-02-22

**Authors:** Mohamed Mokhtar Hefny, Ayman S. Elmezayyen, Ashraf M. Tawfik

**Affiliations:** 1 Engineering Mathematics and Physics Department, Faculty of Engineering and Technology, Future University in Egypt, Cairo, 11835 Egypt; 2 Biological Advanced Materials Laboratory, Physics Department, Faculty of Science, Mansoura University, Mansoura, Egypt; 3 Physics Department, Faculty of Basic Science, New Mansoura University, New Mansoura, Egypt; 4 Theoretical Physics Research Group, Physics Department, Faculty of Science, Mansoura University, Mansoura, 35516 Egypt

**Keywords:** Drug delivery, Statistical physics, Applied mathematics

## Abstract

The need for more efficient drug delivery strategies with ultraprecision and control over the release of drugs has led to the growth of more sophisticated drug-releasing systems as a promising alternative to conventional clinical therapies. This new seed of strategies has explored an encouraging property to overcome the inherent problems of traditional therapies. One of the major challenges for any drug delivery system is the introduction of a complete view of the delivery system. In this article, we intend to elucidate the theoretical proof of concept of the electrosynthesis ATN@DNA core-shell like structure as a model system. Therefore, we present a fractal kinetic model (non-exponential model) taking into consideration the concept of time-dependent diffusion coefficient, which was developed using a numerical method with the help of COMSOL Multiphysics. In addition to that, we present here a general fractional kinetic model in sense of the tempered fractional operator, which leads to better characterized memory properties of the release process. Also, the fractional model is compared with the fractal kinetic model and both offer a good description of drug release processes that present anomalous kinetics. The solutions of the fractal and fractional kinetic models are also fitted successfully with our real-release results.

## Introduction

One of the most popular forms used in pharmaceutical dosage is sphere ensembles introduced as capsules or suspensions for injection or oral administration. Recently, different platforms have been introduced as drug carrier nanomaterials such as metal^1^, nucleic acid^2^, and polymer^3^ based nanoparticles. Most of these attempts were studied to achieve a versatile, controlled, and sustained release delivery system. One of the recently introduced platforms is the structural DNA nanotechnology^1^ which is one of the promising emerged technology extended to numerous fields of applications^2,4^ helping in overcoming most of the present drawback of the current technologies. The technology is based on deoxyribonucleic acid (DNA) biomacromolecule; it has been considered as a probable bioengineering material owing to its biocompatibility, availability, possibly transparent, low toxicity, and biodegradability, etc.^5,6^.

DNA is considered as a polymer of monomers named deoxyribonucleotides, which mainly is a build of a nucleobase- pentose sugar-phosphodiester group^7^. Previously we succussed in the fabrication of a core-shell like structure as a nanocarrier delivery system of atenolol (ATN) drug using electrospherization method, where the core was atenolol, and the shell was DNA^8^. Thus, DNA feasibility in the structural transformation plays an important role in studying drug delivery systems^9,10^. The Kinetics of drug delivery attracted great attention in last years, where it has been studied experimentally and theoretically in different cases^11–13^, which highlights the importance of the chemical kinetics theory in that field.

The chemical kinetics theory is used in many applications in chemistry, physics, biology, biochemistry, etc.^14,15^, it originates from the work of Smoluchowski where he reported that, for homogeneous reactions, the rate constant is proportional to the diffusion coefficient and both of them are time independent in three-dimensional space^16^. However, some processes don’t take place in completely homogeneous conditions such as drug release, where it accrued at the interfaces of different phases as liquid-solid (polymer membrane) boundaries. There are many cases that time independent rate constant can’t be applied and the time-dependent rate constant is only valid such as diffusion-controlled reactions in homogeneous media but with high viscosity and diffusion-controlled reactions in a media with constraints to diffusion near or above the percolation threshold^17,18^. Moreover, the drug release depends significantly on the geometrical characteristics. Therefore, time-dependent rate coefficients (fractal kinetic models) can be used for the study of the drug release process.

Recently, alternative studies have been developed to describe the non-Fickian diffusion of drugs with anomalous kinetic release^19^. Fractional kinetic equations^20,21^ offer an elegant description of experimental data following anomalous diffusion behaviour (non-linear growth of mean square displacement with time, for further details, see Ref.^22^) instead of kinetics problems with fractal kinetic models^23^. For example, the fractional zero and first-order kinetic models have been used to fit the experimental results of in vivo dissolution curves^24^. Also, the fractional pharmacokinetics model has been used to avoid drug accumulation^25^. Moreover, Lenzi et al.^26^ have presented a distributed order fractional model to investigate the drug release profiles of the capsaicinoids-loaded poly microparticles. In addition to that, Kytariolos et al.^27^ present a methodology for power-law in vitro or in vivo (disorder media) by employing fractional calculus formalism. On the other hand, the fractional models of non-Fickian diffusion problems are not exclusive just to drug delivery systems, but it is also widely used in many physical and biological applications such as in Refs.^28,29^.

Using fractional derivatives in physical problems is more sensitive due to the different fractional operators and their physical interpolation. In the literature, the most common use are Caputo’s fractional derivative^30^ and Riemann-Liouville fractional derivative^31^. Sometimes it will be more convenient to use the so-called non-singular fractional derivative such as in Ref.^32^. In this work, we have taken the advantage of results given in^33,34^, where the authors provided the so-called tempered fractional operator that combines non-local and exponential factors. Tempered fractional calculus has been used in many physical situations, especially in the plasma physics^35^, astrophysics^36^, and statistical physics^37^.

According to previous studies, the drug release through a release system can be controlled by various methods such as diffusion, swelling and etc.,^38^. Here, we develop the classical and fractional kinetics models of pharmacokinetics by introducing the tempered fractional diffusion equation. Whereas, the time derivative is replaced by the tempered time-fractional derivative^33^ to capture the natural and sharp cutoff of the drug release profiles. Also, from a physical point of view, the tempered fractional diffusion equation is based on explorative arguments that aim to characterize memory effects during the release mechanism.

## Experimental methodology

**Figure 1:**
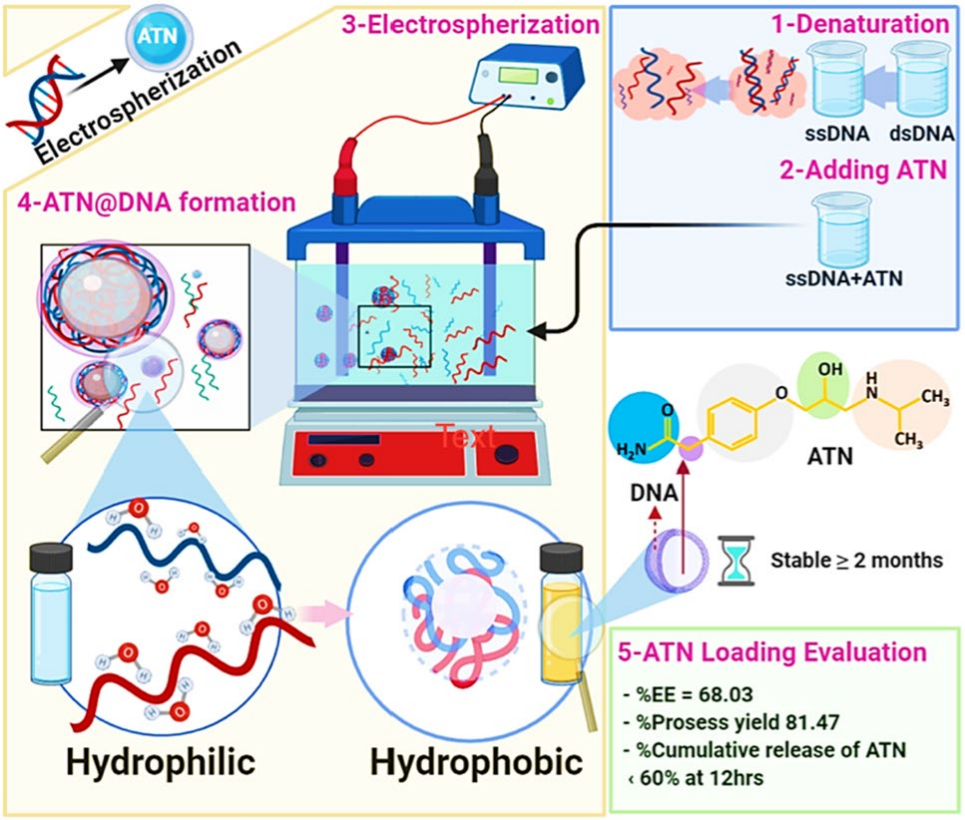
Electro self-assembled ATN@DNA delivery system.

Entrapment of atenolol within DNA nanospheres occurred using our previous method^8^. Briefly, a 0.04 wt.% of ATN was added to 0.2 wt.% of DNA solution prepared by dissolving DNA in deionized bi-distilled water under ambient conditions, then the pH was adjusted to 12 using NaOH. It is named Es ATN@DNA. Two parallel platinum sheets (5 cm apart) were immersed in the alkaline-treated DNA solution as an electrolyte solution, then connected to a potential power supply (ECOS). Electrospherization of DNA molecules was carried out at a constant potential of 2.5 V for 6 h. at ambient conditions under magnetic stirring. In addition, the entrapment efficiency of ATN inside DNA nanospheres for the optimized formulation was 68.03%. In summary, the preparation method of the optimized formulation can be visualized in Fig. 1.

### In vitro releas study

The dialysis bag diffusion method was used to explore the In vitro dissolution findings of the drug delivery system, Es ATN@DNA nanospheres^39^. About 25 mg of lyophilized DNA nanospheres was suspended in (2 mL) phosphate buffer of pH 7.4. The nanospheres were trapped in the dialysis bag with a molecular weight cutoff of 12-14 KDa, tightly closed at both ends, and then carefully immersed in (100 mL) phosphate buffer pH 7.4 filled the receptor compartment. Next, the system was shaken horizontally at 100 strokes per min at a temperature of $370.5^0C$ in a shaking incubator (GFL Gesellschaft für Labortechnik, Burgwedel, Germany). At a certain time interval, the solution outside the dialysis bag was withdrawn (2 ml) using a syringe filter of 0.45 $\mu$m pore size, and a fresh dissolution medium was added to contemporary dissolution media. The ATN drug released from each sample was investigated UV-vis spectrophotometer at a single wavelength of 225 nm against phosphate buffer pH 7.4 as a reference solution. On the other hand, free ATN release was examined under the same conditions. at each time interval, The cumulative percentage drug release was calculated according to the following equation^40^.

1 $$\begin{aligned} C_{c}=C_{B}+\dfrac{V_{s}}{V_{T}} \sum _{B=1}^{n-1} C_{B}. \end{aligned}$$

Where, $C_{c}$ is corrected concentration, $C_{B}$ is base concentration,$V_{s}$ is the volume of sample withdrawn, and $V_{T}$ is the total volume of dissolution.

### In vitro ATN release profile from ATN-loaded nanospheres

**Figure 2:**
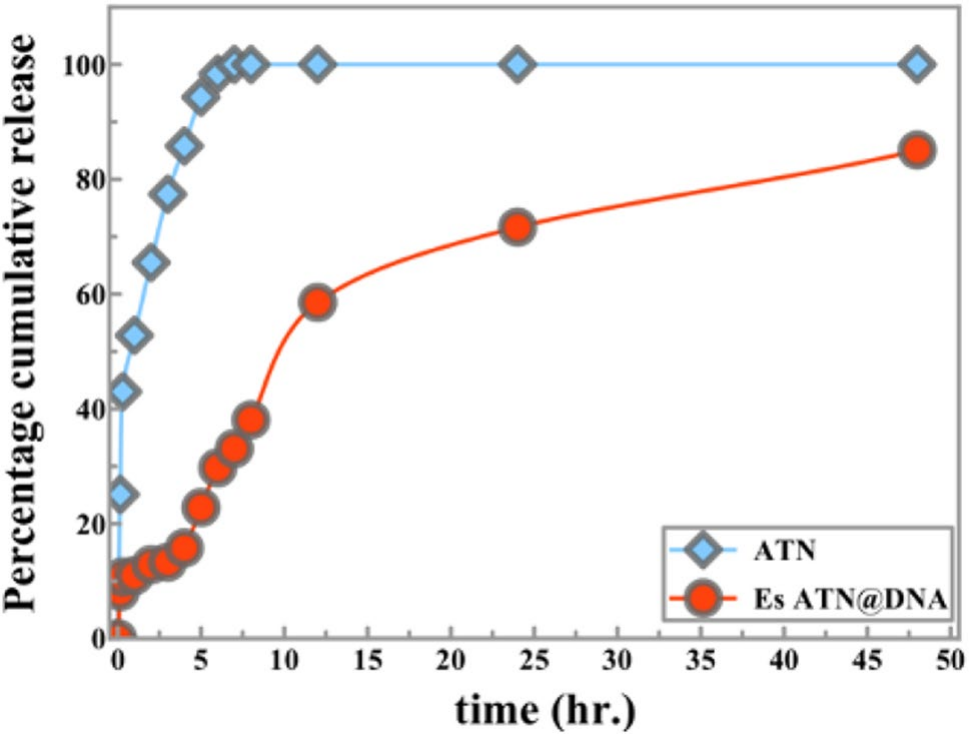
In vitro cumulative release profile of ATN and Es ATN@DNA in phosphate buffer saline pH 7.4.

The process of the drug release from the drug carrier system to the release medium is extensively influenced by many complicated factors such as the morphology of the carrier material, the physicochemical properties of the solute, the release medium, and the probability of the interaction between these entities^41^. In this study DNA biomolecules were introduced to design ATN delivery system with valuable controllable features^42^. The releasing profile of ATN and 0.2 Es ATN@DNA at pH 7.4 for 48 h. depicted in Fig. 2.

In the initial releasing rate within the first 2h, the releasing profiles showed a clear difference between free ATN and 0.2 Es ATN@DNA nanospheres. A fast free ATN release increased consistently over time. After 5 h, 100% cumulative release of ATN was achieved. On the other hand, the release profile of the 0.2EsDNA sample explored a good sustain release behavior with a controlled rate with approximately 60% release at 12 h. The presence of ATN initially deposited near the outer surface of the DNA shell may be the reason for the initial rapid drug release (for more details see Supplementary Table SI.1).

## Fractal kinetic model: numerical investigation of the experimental study

The drug release through the polymer-matrix system can be considered as a diffusion process with geometrical constrains including an infinite sink of the release medium (zero concentration outside the proposed geometry). Therefore, the present model may be described by

2 $$\begin{aligned} \dfrac{\partial c}{\partial t}= \nabla .[D\nabla c] + k_1 c, \end{aligned}$$

where *c* is the drug concentration, *D* is the diffusion coefficient and $k_1$ is the time-dependent reaction rate, which is given by

3 $$\begin{aligned} k_1 = r t^{-h} , \end{aligned}$$

where *r* is a constant and *h* is the so-called fractal kinetics exponent $0\le h<1$. Following the manners used in Refs.^16,25^, the concentration of drug release in the system is given directly through:

4 $$\begin{aligned} \dfrac{\partial c}{\partial t}= k_1 c , \end{aligned}$$

**Figure 3:**
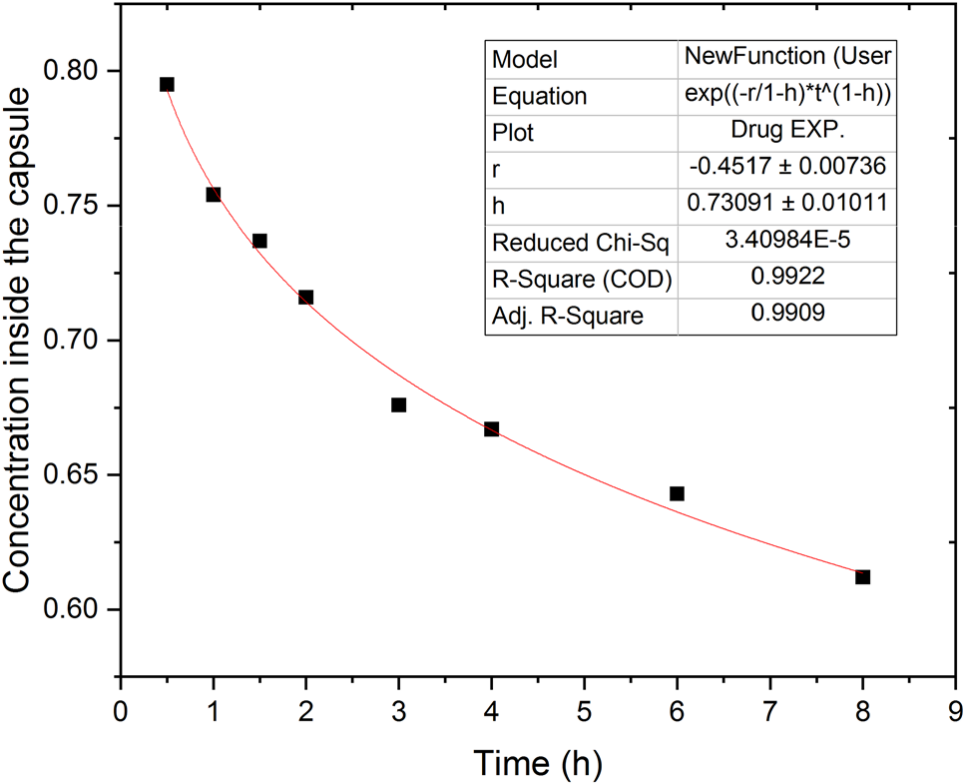
Fitting of the experimentally measured concentration inside the capsule using Eq. 5. The best-fitting parameter values are r=-0.45 and h=0.73.

Then, the accumulated drug concentration in the medium can be presented as^16^:

5 $$\begin{aligned} M = 1-exp({-\alpha t^\beta }), \end{aligned}$$

where *M* is the accumulated drug concentration, $\alpha$ is a scale parameter ($\alpha = -r/(1-h)$) and $\beta$ is a shape parameter ($\beta = 1-h$). Equation 5 is relevant to the so-called Weibull distribution^16^. This type of distribution has a stretched exponential form and it is appropriate to describe the release profile from heterogeneous geometry (fractal and disorder media). Note that, the shaped parameter $\beta$ controls the release profile curve, which can be exponential at $\beta =1$, exponential with a steeper initial slope at $\beta <1$, or S-shaped for $\beta >1$ (For more details see Refs.^16,43,44^). According to that, the concentration of the drug inside the capsule has been fitted using equation 5, see Fig. 3.

We developed a model to study the concentration of the drug over a long time using COMSOL Multiphysics 5.6. COMSOLMultiphysics is a commercial software and it is one of the most powerful tools used to have a real solution to the partial differential Eqs. (PDEs), and the finite element method (FEM) are used to solve the PDE in COMSOLMultiphysics. FEM is the most commonly employed technique for solving problems of engineering and mathematical models, where an approximation of PDEs can be built using different types of discretisations, and then a solution of these PDEs can be obtained numerically. More information about the software and its different interfaces and products can be found elsewhere on^45^.

**Figure 4:**
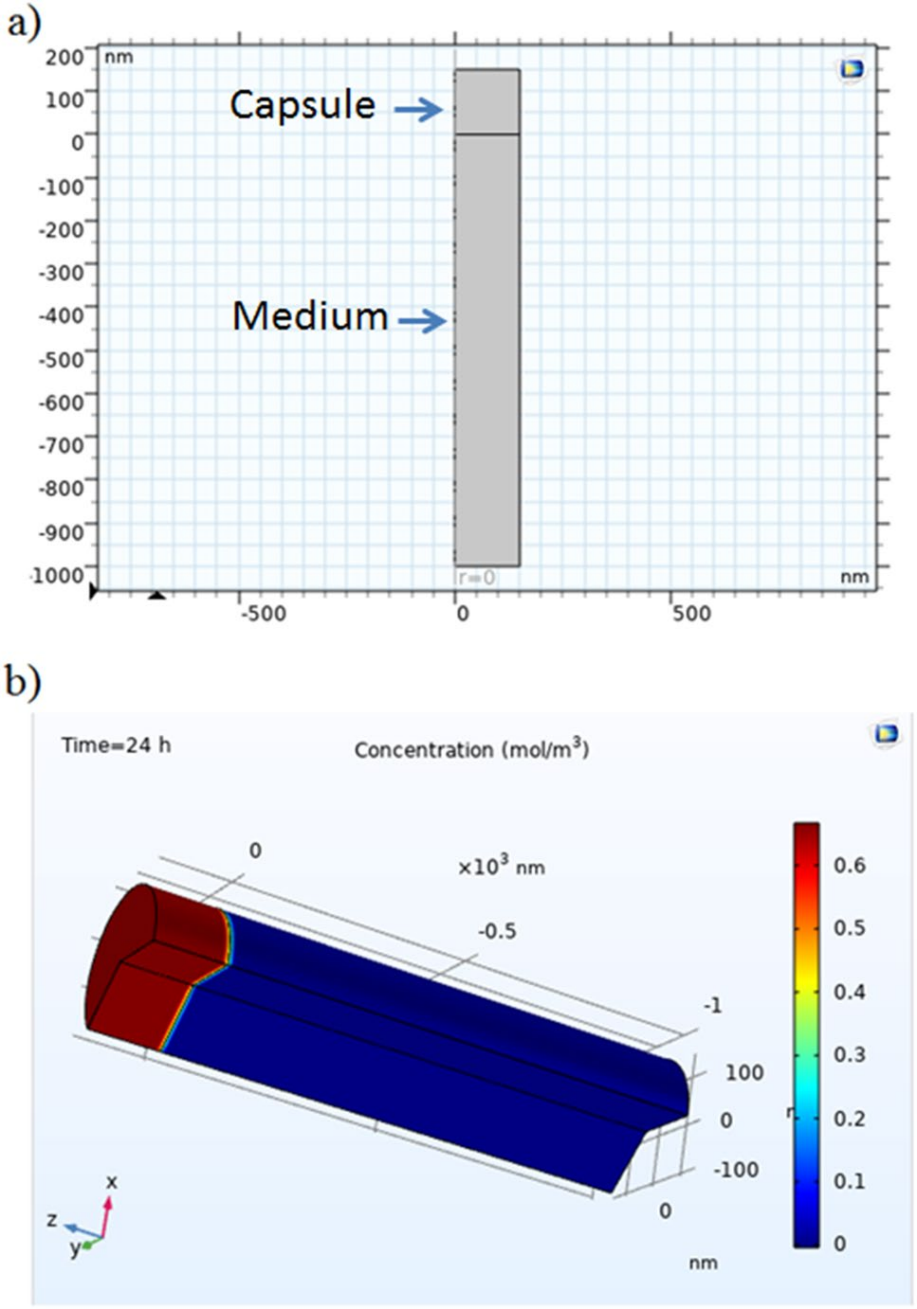
(**a**) The model geometry presents the capsule and the surrounding medium (**b**) a 3D image of the simulated drug concentration inside and outside the capsule after 24 hours.

Transport of diluted species interface was used to solve Eq. 4 using the reaction rate term as described in Eq. 3. A 2D axisymmetric domain was used to simulate the capsule and the surroundings using two rectangles (for simplicity) with lengths of 150 nm and 1000 nm, respectively (see Fig. 4a). Table 1 presents the details of generated grids where 3270 triangle elements and 239 edge elements have been created for our geometry. The initial concentration was 0.8 $mol/m^3$ and it was taken from the experimental results after 0.5-hour and was averaged for three experiments. Therefore, initial concentration was set to 0.8 $mol/m^3$ in the first rectangle, which represents the capsule and it was set to zero in the second rectangle, which represents the medium.

A time dependant problem was studied using Eq. 4 to illustrate the change in the density profile of drug concentration during 24 hours inside and outside the capsule using different values of h (see Eq. (3)). Fig. 4b) illustrates a 3D color-coded map of drug concentration after 24 hours, where it can be clearly seen that most of the drug concentration (around 60$\%$) is still inside the capsule.

**Figure 5:**
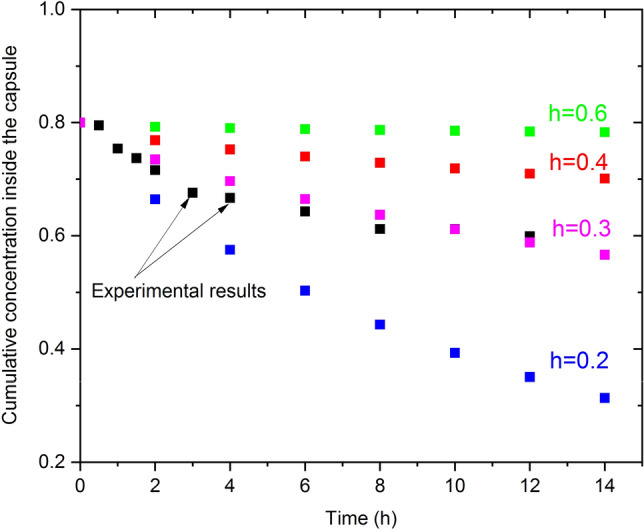
Drug concentration profiles inside the capsule at different times of experimental results and simulated data for different values of h.

**Figure 6:**
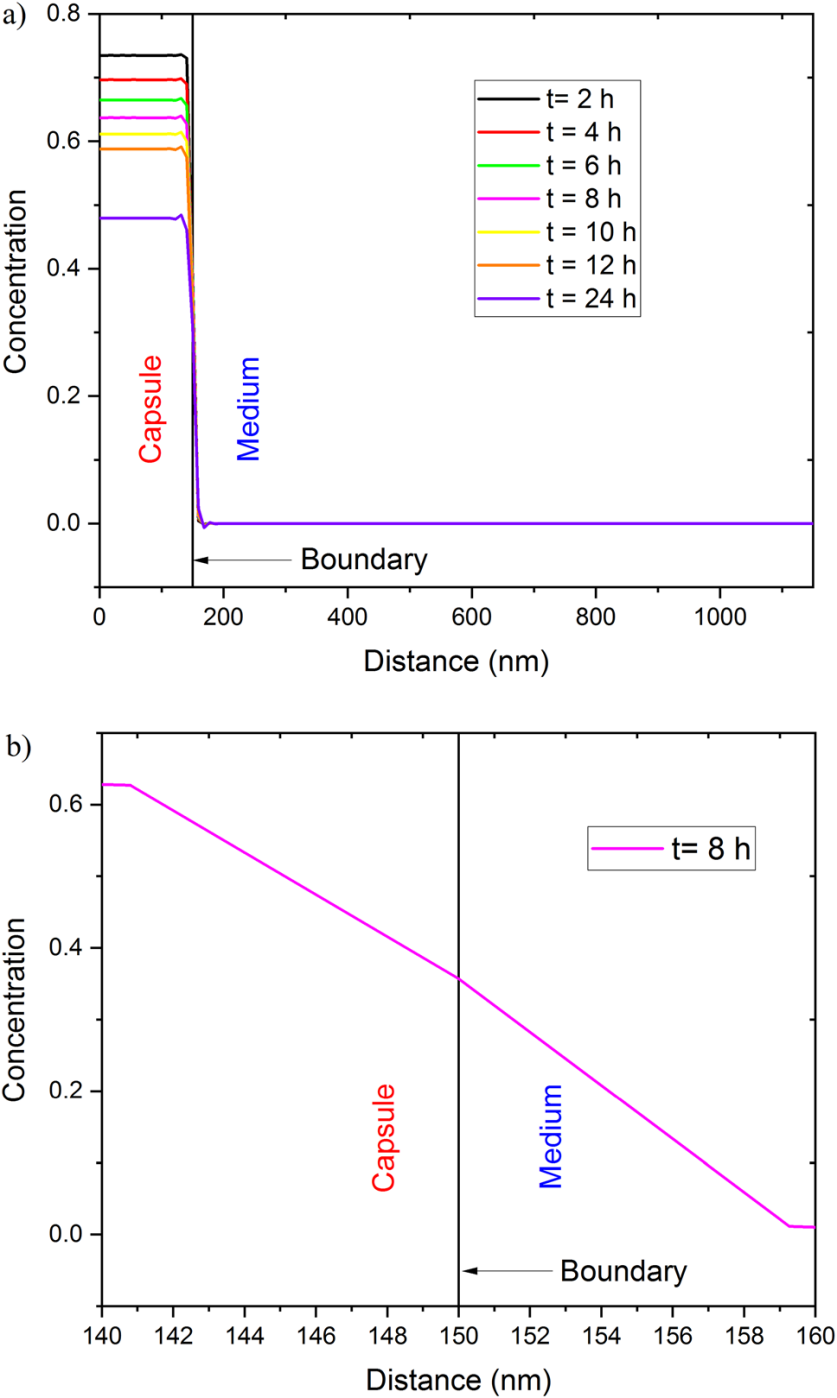
Drug concentration profiles at h = 0.3: (**a**) through the whole geometry of simulation (**b**) around the boundary.

To compare simulation results with the experimental results, the drug concentration profile inside the capsule was drawn versus time for different values of h, see Fig. 5. The simulated results were very close to the experimental ones in the case of h=0.3. For higher values of h, the concentration tends to be almost constant over time, while it decreases significantly with time for lower values of h. The density profile along the whole geometry is shown in Fig. 6a for different values of time to study the drug behavior over time inside and outside the capsule. It can be seen clearly from Fig. 6a that the concentration decreases with time to about 60$\%$ of the initial value after 24 hours inside the capsule (40$\%$ outside the capsule as measured experimentally). It can also be seen that the main drop in the concentration occurred near the boundary, see Fig. 6b inside and outside the capsule, which is another piece of evidence that the simulation is very close to the experimental work.

**Table 1:** Element statistics of the used mesh in our numerical model.

Element type	Number
Triangles	3270
Mesh vertices	1749
Edge elements	239
Vertex element	6
Number of elements	3270
Element area ratio	0.4085
Mesh area	172500 $nm^2$
Average element quality	0.9553
Minimum element quality	0.6831

## Tempered fractional kinetic model: analytical investigation of the experimental study

The Tempered fractional derivatives have many advantages in mathematical models, especially to capture the cutoff in real physical systems. Also the tempered operator can used to exhibit a semi-long rang dependence with combines the non-locality and weak singularity^34^. In the frameworks of non-Fickian diffusion problems, the tempered fractional diffusion equations have been arises in different situations for more details see Refs.^46,47^.

According to previous motivations of using the tempered fractional operator, the drug release through the polymer-matrix system can be considered a diffusion process with geometrical constraints. In this part, this will be studied analytically by considering the so-called tempered fractional reaction-diffusion equation, which can be written as follows:

6 $$\begin{aligned} D_{t}^{\alpha ,\lambda } c(x,t)= \kappa _{\alpha } \frac{\partial ^2}{\partial x^2} c(x,t)-R c(x,t), \end{aligned}$$

with initial and boundary values given by $c(x,0)=\delta (x)$ and $c(\pm \infty ,t)=0$. Where $\kappa _{\alpha }$ is the diffusion coefficient and *R* represents the reaction rate coefficient, which makes sense to capture the mechanism. The fractional derivative in the LHS of Eq. (6) is the tempered fractional operator in sense of Caputo’s fractional derivative^33,48^, which is defined as

7 $$\begin{aligned} D_{t}^{\alpha ,\lambda } f(t)=e^{-\lambda t} * D_{t}^{\alpha } \left( e^{\lambda t} f(t) \right) , \end{aligned}$$

where, $\lambda \ge 0$ is the so-called tempered parameter, $0<\alpha <1$ is the fractional order of derivative and $D_{t}^{\alpha }$ is the regular Caputo’s fractional derivative for^49^.

Applying the Laplace transform to Eq. (6), we obtain

8 $$\begin{aligned} (s+\lambda )^{\alpha } c(x,s)-(s+\lambda )^{\alpha -1}=\kappa _{\alpha } \frac{\partial ^2}{\partial x^2} c(x,s)-R c(x,s), \end{aligned}$$

where the Laplace transform of tempered fractional Caputo’s derivative in case of $0<\alpha <1$ is given by

9 $$\begin{aligned} \mathscr {L} \left( D_{t}^{\alpha ,\lambda } g(t) \right) = (s+\lambda )^{\alpha } g(s)-(s+\lambda )^{\alpha -1}. \end{aligned}$$

And by applying the Fourier transform to the above equation, we obtain the density in the Laplace-Fourier domain

10 $$\begin{aligned} c(k,s)=\dfrac{(s+\lambda )^{\alpha -1}}{(s+\lambda )^{\alpha }+\kappa _{\alpha } k^{2}+R}. \end{aligned}$$

To measure the concentration of drugs that is present in the system ( the so-called survival probability), we use the definition^50^

11 $$\begin{aligned} P(t)=\int _{-\infty }^{\infty } c(x,t)= \mathscr {F} \left( c \right) _{k=0}. \end{aligned}$$

Through Eqs. (10 and 11), on can obtain the following expression

12 $$\begin{aligned} P(t)=\mathscr {L}^{-1} \left( \dfrac{(s+\lambda )^{\alpha -1}}{(s+\lambda )^{\alpha }+R} \right) =e^{-\lambda t} E_{\alpha } (-Rt^{\alpha }), \end{aligned}$$

where $E_{\gamma } \left( y \right)$ is the Mittag-Leffler function given by Eq. (A.1), which is used especially to describe processes following power-law densities.

Therefore, the amount of drug release (quantity of substance present in the aqueous medium)) is equal

13 $$\begin{aligned} \sigma (t)=\sigma _{0} \left( 1-e^{-\lambda t} E_{\alpha } (-Rt^{\alpha }) \right) , \end{aligned}$$

where $\sigma _{0}$ is the parameter corresponding to the plateau of the dissolution curve. Also, note that Eq. (13) is a direct solution of the tempered fractional kinetic equation given in the form

14 $$\begin{aligned} D_{t}^{\alpha ,\lambda } c(t)= -R c(t). \end{aligned}$$

The above equation represents the tempered fractional kinetic model corresponding to the so-called first-order kinetic model. For more analysis of drug release in a polymer matrix system, we can calculate the mean square displacement through Eq. (10), which is given by:

15 $$\begin{aligned} \langle x^2(t)\rangle =\mathscr {L}^{-1} \left[ -\frac{\partial ^{2}c(k,s)}{\partial k^{2}} \right] _{k=0}=2 \kappa _{\alpha }t^{\alpha } e^{-\lambda t}E_{\alpha ,\alpha +1}^{2} (-Rt^{\alpha }), \end{aligned}$$

where $E^{n}_{a,b}\left( z \right)$ is the so-called three-parameter Mittag-Leffler function defined in Eq.(A.2).

**Figure 7:**
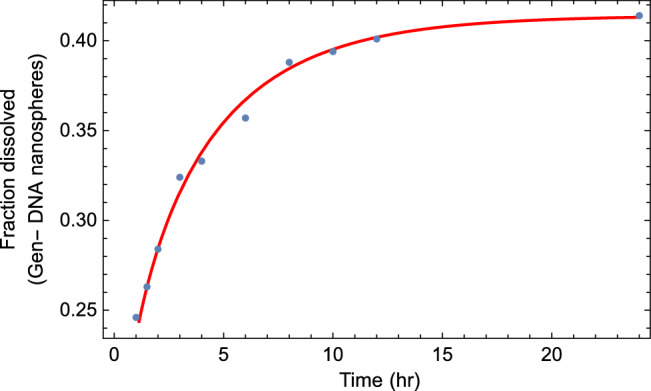
Plot corresponding to the data sets of in vitro Gen-DNA nanospheres with the fitted curve of Eq. (13). The best fitting parameter values are $\alpha =0.33$, $R=0.75$, $\lambda =0.2$ and $\sigma _{0}=0.414$.

**Figure 8:**
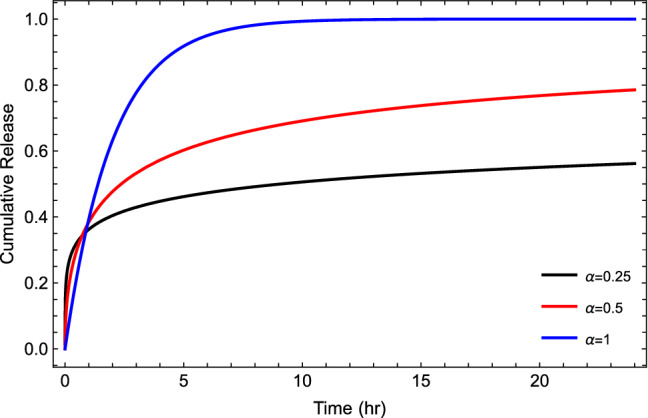
Plot of cumulative drug release given by Eq. (13) for different values of fractional orders $\alpha$ at $\lambda =0$ and $R=0.5$.

**Figure 9:**
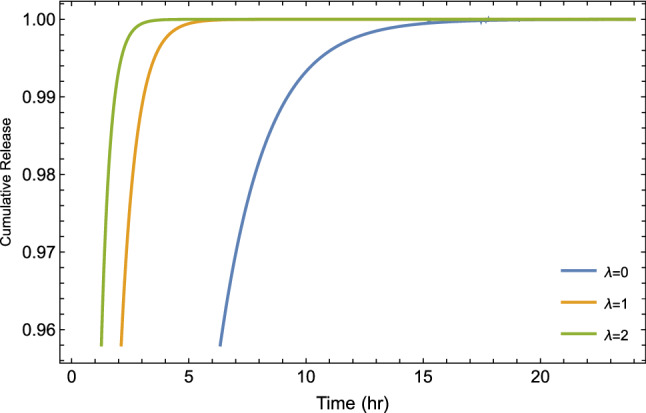
Plot of cumulative drug release given by Eq. (13) for different values of tempered parameter $\lambda$ at $\alpha =1$ and $R=0.5$.

**Figure 10:**
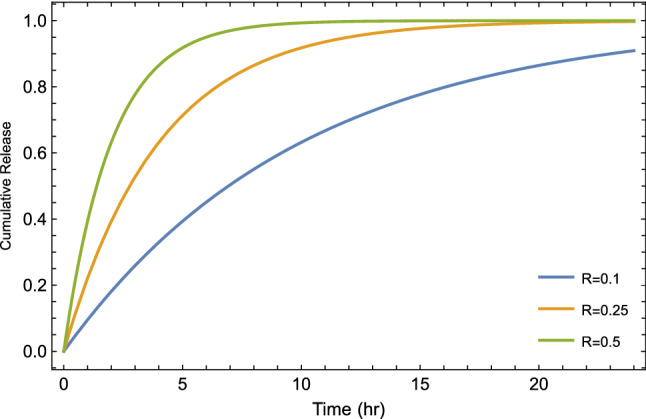
Plot of cumulative drug release given by Eq. 13 for different values of reaction constant at $\alpha =1$ and $\lambda =0$ (refer to the classical first-order kinetic model with different constant rates).

**Figure 11:**
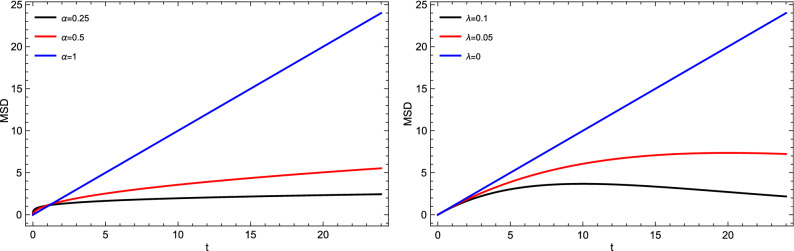
Mean square displacement Eq. 15 for different values of (**a**) time-fractional order $\alpha$ and (**b**) tempered parameter in case of $R=0$ and $\kappa =0.5$.

**Figure 12:**
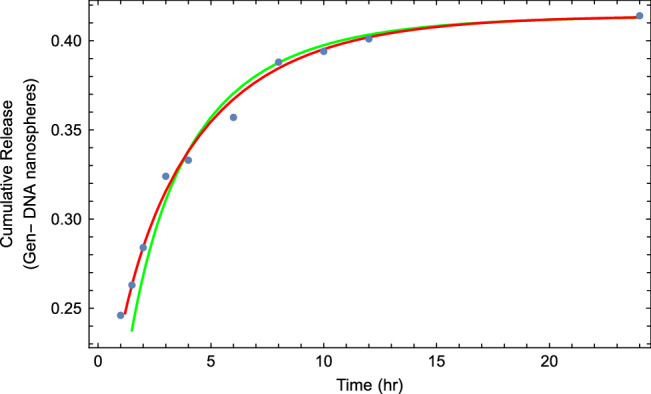
The fitting of experimental drug release data with the fractal kinetic model (green curve) and tempered fractional kinetic model (red curve).

The tempered fractional model represents a mathematical generalization of commonly used models based on variant parameters ($\alpha$ and $\lambda$). In general, the fractional order $\alpha$ envisages the degree of heterogeneity and is attributed physically to sub-diffusive motion. Also, we can show the correlation of the so-called tempered parameter $\lambda$ in the time estimate to form a gel layer during the release process. In general, we can see the effect of the three main parameters introduced in our fractional model on the behavior of the drug release via Fig 7 to Fig. 12.

In Fig. 7, we show the fitting curve for release profiles of ATN-DNA nanosphere via the tempered fractional kinetic models. Our illustration is based on choosing an arbitrary value of the time-fractional order ($\alpha =0.33$), tempered parameter ($\lambda =0.2$), and reaction rate ($R=0.75$).

Figure 8 illustrates the cumulative drug release for different values of the time-fractional order, which reflects as a control parameter in the saturation value. It seems that the release in the subdiffusive case ($\alpha =0.25, 0.5$) is actually faster than the normal case ($\alpha =1$) at very short times and starts to lag behind the normal case. In addition to that, Fig. 9, shows the cumulative drug release in case of different values of tempered parameters $\lambda$. From Fig. 9, we can find that increasing the value of $\lambda$ decreases the time indeed to reach the saturation level, which means short diffusion length and then a long time for the system to form a gel layer. Furthermore, In Fig. 10, as an illustration, we show the change of cumulative release with different values of reaction constant *R*. On the other side, to show and track the drug molecules inside the capsule, we illustrate the mean square displacement in Fig. 11 for different time-fractional order $\alpha$ and tempered parameter $\lambda$ in case of neglect the sink term. From Fig. 11, we show that decreasing values of time-fractional order mean slower movement of drug particles. However, decreasing the value of the tempered parameter capture the normal diffusion processes in case of zero reaction rate.

## Conclusion

To wrap things up, we combined the fractal (time-dependent coefficient rate) and tempered fractional kinetic models with the real drug release data in Fig. 12, which indicates that the two models are equally suitable for describing drug release profiles. Practically, the fractional model performs a little better than the fractal model according to our release device. However, the fractal model is still easier to use. But the good result is both models are reliable in the description of the final stages of release processes. Moreover, they are reliable to estimate the particle remaining in the core-shell. These findings can help us in the future to understand and predict the behavior of the drug release process for different types of drugs and media for short and long times, which reduces the number of experiments and saves time and effort while giving the same results.

## Data availability

The data presented in this study are available on request from the corresponding author and its supplementary information file.

### Supplementary Information

Supplementary Information.

## Appendix: Mittag-Leffler functions

The Mittag-Leffler function of the first kind is defined as^51^

A.1 $$\begin{aligned} E_{\gamma } \left( y \right) =\sum \limits _{k=0}^{\infty }\frac{y^{k}}{\Gamma (\gamma k+1)}. \end{aligned}$$

The three-parameter Mittag-Leffler function is defined as^52^

A.2 $$\begin{aligned} E^{n}_{a,b}\left( z \right) =\sum \limits _{k=0}^{\infty } \frac{ \Gamma (n+k)}{ \Gamma (n)} \frac{ z^{k}}{\Gamma (a k+b) k!}. \end{aligned}$$
