# Supplementary material for: Anomalous kinetic study of atenolol release from ATN@DNA a core-shell like structure
Source: Sci Rep. 2023 Feb 22;13:3120. doi: 10.1038/s41598-023-29774-8 (PMC9947108; doi:10.1038/s41598-023-29774-8)
Supplement: Supplementary file 1 — Supplementary Information. [file 41598_2023_29774_MOESM1_ESM.pdf]

Anomalous kinetic study of atenolol release from ATN@DNA a core-shell like structure

Mohamed Mokhtar Hefny, Ayman S. Elmezayyen, and Ashraf M. Tawfik*

Supplementary Table

Table SI.1: In vitro cumulative release data of ATN and Es ATN@DNA

Time (hr)	Drug alone			Gen- DNA nanospheres		
0.5	38.5	34.1	39.3	21.3	19.8	20.4
1	52.4	48.8	49.1	25.6	23.8	24.4
1.5	65.2	55.8	65.9	27.7	25.9	25.3
2	71.2	68.8	70.6	30.3	26.4	28.5
3	80.2	74.8	78.4	35.6	30.1	31.5
4	85.6	77.9	83.4	36.2	31.2	32.5
6	86.8	80.4	86.7	38.4	33.5	35.2
8	93.2	87.9	87.4	40.1	34.8	41.5
10	94.4	90.2	87.8	42.3	38	36.1
12	95.6	92.1	88.4	43.3	39.8	37.2
24	97.8	93.2	95.8	45.5	40.1	38.8
48	100.2	96.4	98.8	45.7	42	39.7
